# Hospitalizations and Deaths Caused by Methicillin-Resistant *Staphylococcus aureus*, United States, 1999–2005

**DOI:** 10.3201/eid1312.070629

**Published:** 2007-12

**Authors:** Eili Klein, David L. Smith, Ramanan Laxminarayan

**Affiliations:** * Resources for the Future, Washington DC, USA; † National Institutes of Health, Bethesda, Maryland, USA

**Keywords:** Methicillin-resistant, Staphylococcus aureus infections, hospitalization trends, costs, pneumonia, septicemia, community-associated, vancomycin, cellulitis, research

## Abstract

Hospital-acquired infections with *Staphylococcus aureus*, especially methicillin-resistant *S. aureus* (MRSA) infections, are a major cause of illness and death and impose serious economic costs on patients and hospitals. However, the recent magnitude and trend of these infections have not been reported. We used national hospitalization and resistance data to estimate the annual number of hospitalizations and deaths associated with *S. aureus* and MRSA from 1999 through 2005. During this period, the estimated number of *S. aureus*–related hospitalizations increased 62%, from 294,570 to 477,927, and the estimated number of MRSA-related hospitalizations more than doubled, from 127,036 to 278,203. Our findings suggest that *S. aureus* and MRSA should be considered a national priority for disease control.

## Abstract 2

MRSA should be a national priority for disease control.

*Staphylococcus aureus* is a leading cause of hospital-acquired infections. It is the primary cause of lower respiratory tract infections and surgical site infections (*1*,*2*) and the second leading cause of nosocomial bacteremia (*3*), pneumonia, and cardiovascular infections (*1*,*2*). Infections with *S*. *aureus* are especially difficult to treat because of evolved resistance to antimicrobial drugs. Resistance to penicillin and newer narrow-spectrum β-lactamase–resistant penicillin antimicrobial drugs (e.g., methicillin, oxacillin) appeared soon after they were introduced into clinical practice in the 1940s and 1960s, respectively (*4*). Penicillin resistance was initially confined to a small number of hospitalized patients, but resistance spread as use of penicillin increased, first to other hospitals and then into the community (*5*). By the late 1960s, >80% of community- and hospital-acquired *S*. *aureus* isolates were resistant to penicillin (*4*). Recent reports suggest that the evolution and spread of methicillin-resistant *S*. *aureus* (MRSA) seems to be following a similar wavelike emergence pattern (*5*).

MRSA is now endemic, and even epidemic, in many US hospitals, long-term care facilities (*6*), and communities (*7*,*8*). Contrary to the generally accepted view, community-associated MRSA strains may be spreading into the healthcare system rather than the other way around (*9*). Data from the National Nosocomial Infections Surveillance system suggest that in intensive care units the proportion of *S*. *aureus* isolates that are resistant to methicillin has increased to 59.5%–64.4% (*10*,*11*). Recent reports also suggest that community-associated MRSA infections have become the dominant cause of community-associated *S*. *aureus* skin and soft tissue infections (*9*,*12*). An understanding of the magnitude of the problem requires accurate national estimates of incidence. However, national studies examining the effect of *S*. *aureus* or MRSA on the healthcare system are >5 years old (*13*,*14*). For 2000–2001, Noskin et al. estimated that there were 290,000 *S*. *aureus*-related hospitalizations (*14*). Kuehnert et al. estimated a similar number of *S*. *aureus*–related hospitalizations for 1999–2000 and reported that 43.2% (125,969) were likely resistant to methicillin (*13*).

In this study, we estimated the magnitude of the effect and trend in the incidence and associated mortality rates of infections related to *S*. *aureus* and MRSA over a 7-year period, from 1999 through 2005, paying particular attention to the overall *S*. *aureus* infection level and the trend of typical community-associated infections. Evidence on the magnitude and trend of the problem on a national level informs rational, evidence-based decisions about how to allocate resources and adjust healthcare policy to address this issue. Infection trends are useful to clinicians, hospital administrators, insurers, and policymakers who make decisions regarding control measures, especially infection-control measures to contain the spread of nosocomial and community-associated pathogens.

## Methods

Our analysis focused on the period 1999–2005 and followed an approach similar to that described by Kuehnert et al. (*13*). Estimated incidence of *S*. *aureus* was based on hospitalizations with *S*. *aureus*–related discharge diagnoses from the National Hospital Discharge Survey (NHDS). The NHDS covers ≈270,000 patients and 500 short-stay hospitals by using a stratified, multistage survey to create a nationally representative annual sample of discharge records. Children and general hospitals are included; federal, military, Veterans Affairs, or institutional hospitals are not included. Each discharge record contains <7 different International Classification of Diseases, Ninth Revision (ICD-9), Clinical Modification, discharge diagnosis codes and is population weighted on the basis of the probability of sample selection and adjusted for nonresponse. All acute-care hospitalizations, excluding those of infants born in the hospital, were considered.

*S*. *aureus*–related discharges were included if any of the 7 diagnosis codes contained specific *S*. *aureus* infection codes: 038.11 (*S*. *aureus* septicemias), 482.41 (*S*. *aureus* pneumonias), and 041.11 (other *S*. *aureus* infections). Records that contained multiple *S*. *aureus*–related discharge codes were only counted once, with septicemia preferentially included, followed by *S*. *aureus*–related pneumonia.

Because there is no MRSA-specific ICD-9 code, we indirectly estimated the proportion of *S*. *aureus*–related infections that were methicillin resistant by using antimicrobial drug testing data from The Surveillance Network (TSN) Database-USA (Focus Diagnostics, Herndon, VA, USA). TSN is an electronic repository of susceptibility test results collected from >300 microbiology laboratories in the United States; it has been used extensively to evaluate antimicrobial drug resistance patterns and trends (*15*). Participating laboratories are geographically dispersed and make up a nationally representative sample on the basis of hospital bed size and patient population. Patient isolates are tested for susceptibility to several different antimicrobial agents on site as part of routine diagnostic testing by using standards established by the National Committee for Clinical Laboratory Standards (NCCLS) and approved by the US Food and Drug Administration (*15*). Results are filtered to remove repeat isolates and identify microbiologically atypical results for confirmation or verification before being included.

We included *S*. *aureus* isolates from inpatient areas that were tested for susceptibility to oxacillin (which is used as a proxy for all β-lactam antimicrobial drugs, including methicillin) and classified as susceptible, intermediate, or resistant according to NCCLS breakpoint criteria. Data included >65,000 isolates annually, of which <0.01% had intermediate resistance and so were classified susceptible. To ensure comparability with NHDS data, isolates were stratified by the type of infection (i.e., isolates from the lungs were classified as pneumonias; those from the blood, as septicemias or bacteremias) and geographic region based on the US Census Bureau regions.

The annual estimated number of *S*. *aureus*–related hospitalizations was obtained from NHDS. The total number of MRSA-related hospitalizations was estimated by multiplying the number of *S*. *aureus*–related infections by the estimated percentage of *S*. *aureus* isolates that were resistant, stratified by infection type and region. Frequencies of primary and secondary diagnoses were also extracted for all hospitalizations that included *S*. *aureus*–related infections.

Relative standard errors for incidence of *S*. *aureus* were calculated by following guidelines for NHDS accuracy described by Dennison and Pokras (*16*). Standard errors and 95% confidence intervals (CIs) were calculated by multiplying the relative standard error by the estimated incidence. CIs for TSN data were calculated by using the Wilson score method and incorporating continuity correction as detailed by Newcombe (*17*). The variance of MRSA incidence was estimated by using the method described by Barnett (*18*) and Goodman (*19*).

NHDS reports whether or not hospitalization results in patient death but does not specify the cause of death. Because the primary diagnosis suggests that the disease played a role in patient death, we estimated the number of *S*. *aureus*–related deaths where the primary diagnosis code was an *S*. *aureus*–related code. We used the same procedure as described above to determine the estimated number of deaths for which MRSA was involved.

## Results

From 1999 through 2005, annual hospital discharges in the United States increased ≈8%, from 32.1 million to 34.7 million. During this period, the estimated number of hospitalizations involving *S*. *aureus*–related infections increased 62%, from 294,570 (95% CI 257,304–331,836) to 477,927 (95% CI 421,665–534,189). *S*. *aureus*–related hospitalizations with diagnosis codes for septicemia and pneumonia increased 38% and 7%, respectively, and hospitalizations involving other *S*. *aureus*–related infections in conditions classified elsewhere nearly doubled. Overall, the rate of *S*. *aureus*–related diagnoses per 1,000 hospitalizations increased 50%, from 9.17 to 13.79 (Table 1).

**Table 1:** *Staphylococcus aureus* and methicillin-resistant *S. aureus* (MRSA)–related hospital discharge diagnoses, by infection site and year, United States

Discharge diagnosis	1999	2000	2001	2002	2003	2004	2005
All discharges	32,131,876	31,705,672	32,652,588	33,726,612	34,738,412	34,864,168	34,667,316
*S. aureus* septicemias	75,125	73,206	77,998	82,813	92,247	92,785	103,300
% MRSA	41	45	48	49	52	54	54
MRSA septicemias	31,044	33,251	37,381	40,197	47,745	50,238	56,248
*S. aureus* pneumonias	58,833	53,692	63,759	64,294	58,511	71,275	63,185
% MRSA	52	54	56	58	58	59	58
MRSA pneumonias	30,632	29,210	35,893	37,120	33,965	41,988	36,540
Other *S. aureus* infections	160,612	161,614	189,715	211,310	245,971	272,873	311,442
% MRSA	41	44	48	50	54	58	60
Other MRSA infections	65,361	71,048	90,163	106,174	132,154	158,211	185,415
Total *S. aureus* infections	294,570	288,512	331,472	358,417	396,729	436,933	477,927
Overall % MRSA	43	46	49	51	54	57	58
Total MRSA infections	127,036	133,510	163,437	183,491	213,864	250,438	278,203

**Figure 1:**
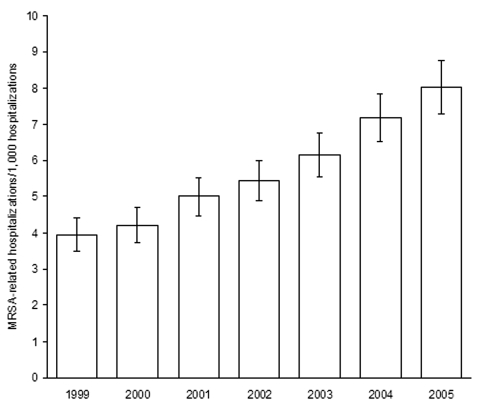
Estimated methicillin-resistant *Staphylococcus aureus* (MRSA)–related hospitalization rates, United States, 1999–2005. Rates are no. MRSA-related discharges/1,000 hospitalizations. Error bars represent 95% confidence intervals.

From 1999 through 2005, estimated MRSA-related hospitalizations more than doubled, from 127,036 (95% CI 112,356–141,716) to 278,203 (95% CI 252,788–303,619). MRSA-related hospitalizations with a diagnosis code for septicemia increased 81.2%, from 31,044 (95% CI 25,170–36,918) to 56,248 (95% CI 46,830–65,665), and MRSA-related hospitalizations with a diagnosis code for pneumonia increased 19.3%, from 30,632 (95% CI 24,597–36,666) to 36,540 (95% CI 29,527–43,554). The largest increase in MRSA-related hospitalizations involved infections outside the lungs or blood; these almost tripled from 65,361 (95% CI 55,801–74,920) to 185,415 (95% CI 162,102–208,728). Overall, the rate of MRSA-related discharges per 1,000 hospitalizations more than doubled, from 3.95 to 8.02 (Figure 1).

**Figure 2:**
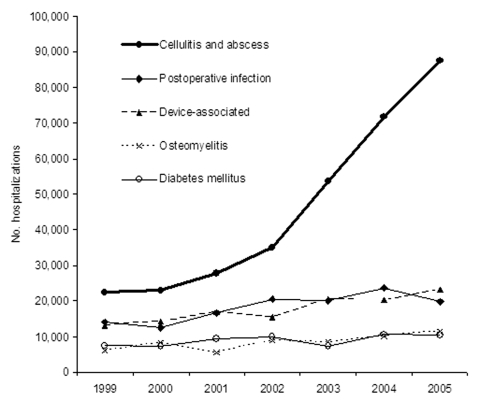
Primary diagnoses of *Staphylococcus aureus*–related hospitalizations. The most frequent primary diagnosis associated with other *S. aureus*–related infections was other cellulitis and abscess (International Classification of Diseases [ICD]-9 682), followed by postoperative infection (ICD-9 998.59), infections from an implanted device or graft (ICD-9 996), osteomyelitis (ICD-9 730), and diabetes mellitus (ICD-9 250). Cellulitis and abscess infections increased at a rate >25% per year from 1999 through 2005. No other primary diagnosis infection showed a major increase over this period.

In hospitalizations for which *S*. *aureus*–related septicemia and pneumonia were listed as any 1 of the 7 discharge diagnoses, these diagnoses were coded as the primary diagnosis, on average, in 38% (standard deviation 6.4%) and 54% (3.7%) of records, respectively, over the 7-year period. The most frequent primary diagnosis associated with other *S*. *aureus*–related infections was other cellulitis and abscess (ICD-9 682), followed by postoperative infection (ICD-9 998.59), infections from an implanted device or graft (ICD-9 996), osteomyelitis (ICD-9 730), and diabetes mellitus (ICD-9 250). Cellulitis infections increased >25% per year from 22,451 (95% CI 17,007–27,895) to 87,500 (95% CI 75,485–99,515), which was nearly a 4-fold increase. No other primary diagnosis infection code increased over this time period (Figure 2).

Similar rates of discharge associated with *S*. *aureus*–related and, more specifically, MRSA-related infections per 1,000 hospitalizations were observed across all 4 US regions (Northeast, South, Midwest, and West; Table 2). Overall, the rate of *S*. *aureus*–related infections increased 5% per year in the Northeast, 7% in the Midwest and South, and 8% in the West. The rate of MRSA-related infections in the Northeast, Midwest, and South increased 9%, 11%, and 12% per year, respectively. In contrast, the West had the lowest incidence and frequency of MRSA-related infections, but the rate of MRSA-related infections increased 18% per year. Although increases were considerable, none of the rates in any region was significantly different in any year from the others at the 95% CI level.

**Table 2:** Hospitalizations and rates of infections with *Staphylococcus aureus* and methicillin-resistant *S. aureus* (MRSA) by region and year, United States*

Region	1999	2000	2001	2002	2003	2004	2005
Northeast	8.42 (3.58)	8.61 (3.9)	10.01 (4.9)	10.62 (5.22)	11.25 (5.65)	11.07 (5.84)	11.59 (6.12)
Midwest	8.53 (3.84)	9.59 (4.53)	9.8 (4.84)	9.33 (4.8)	9.65 (5.04)	11.29 (6.54)	12.47 (7.23)
South	9.71 (4.63)	9.44 (4.68)	10.14 (5.33)	11.17 (6.15)	12.5 (7.25)	13.46 (8.21)	14.77 (9.31)
West	9.75 (3.15)	8.33 (3.14)	10.85 (4.61)	11.05 (4.98)	11.57 (5.87)	13.75 (7.39)	15.84 (8.55)

*Rates are no. hospitalizations with *S. aureus* MRSA–related discharge diagnoses/1,000 discharges. Values in parentheses are rates for MRSA.

**Figure 3:**
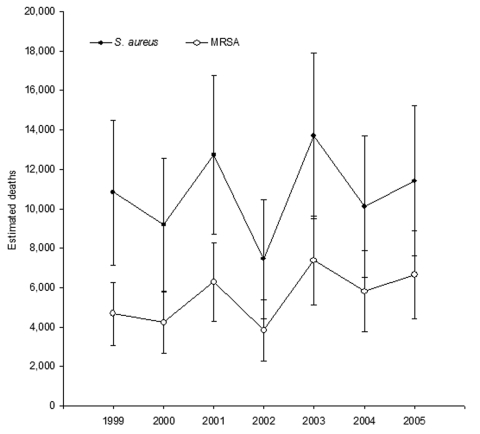
Estimated hospital deaths associated with *Staphylococcus aureus* and methicillin-resistant *S. aureus* (MRSA), United States, 1999–2005. Error bars represent 95% confidence intervals.

In 2005, there were ≈11,406 *S*. *aureus*–related deaths (95% CI 7,609–15,203), of which 6,639 were MRSA-related (95% CI 4,429–8,850). Since 1999, no trend was seen in the number of deaths. We estimated that *S*. *aureus*–related deaths averaged ≈10,800 per year (range 7,440–13,676) and MRSA-related deaths averaged ≈5,500 per year (range 3,809–7,372) (Figure 3). However, the percentage of *S*. *aureus*–related and MRSA-related hospitalizations that resulted in death did show a trend, a decrease from ≈3.7% in 1999 to only 2.4% in 2005. We also calculated the number of deaths in which any diagnosis code was *S*. *aureus*–related. These calculations showed that deaths with an *S*. *aureus*–related discharge code increased 18% from 24,715 (95% CI 17,853–31,577) to 29,164 (95% CI 21,620–36,708) from 1999 through 2005. Deaths in which MRSA was likely present increased >50%, from 11,240 (95% CI 8,117–14,362) to 17,260 (95% CI 12,794–21,726) over the same period. However, despite the increases, the percentage of *S*. *aureus*–related hospitalizations that resulted in death decreased from 8.4% in 1999 to 6.1% in 2005, and the percentage of MRSA-related hospitalizations that resulted in death decreased from 8.8% to 6.2%.

*S*. *aureus* resistance to ampicillin/sulbactam, cephalothin, and erythromycin increased 21%, 35%, and 27%, respectively, during the study period. Resistance to gentamicin and trimethoprim-sulfamethoxazole decreased 76% and 64%, respectively. No instances of vancomycin-resistant (or intermediate-resistant) *S*. *aureus* in hospitalized patients were reported.

## Discussion

MRSA, a common cause of nosocomial infections, has emerged as an increasingly common cause of community-associated infections (*20*). Our analysis extends the work of Kuehnert et al. (*13*) and quantifies recent trends and the effect of *S*. *aureus* and MRSA on the US healthcare system.

This study focused on the effect and trends in the incidence of *S*. *aureus*–related infections generally and MRSA-related infections specifically. Although the number of hospitalizations associated with an *S*. *aureus* infection increased 62% or ≈8.4% per year, the number of *S*. *aureus* infections resistant to methicillin increased 119% or ≈14% per year. In addition, although steady growth was observed in the incidence of *S*. *aureus*– and MRSA-related septicemia, pneumonia, and device-associated infections that are typically nosocomial, dramatic increases were observed in the incidence of skin and soft tissue infections that are typically community associated. We also found no trend in the number of deaths caused by MRSA, and a decreasing trend in the percentage of *S*. *aureus*– and MRSA-related hospitalizations that resulted in death. These results suggest a change in the ecology of the disease; community-associated MRSA is spreading more rapidly and possibly making its way into hospitals.

The indication that community-associated MRSA is spreading rapidly into hospitals has implications for hospital and community infection control as well as empirical treatment. In hospitals, handwashing practices, which have been shown to be the leading intervention for limiting the spread of nosocomial infections, should be improved to meet recommended guidelines (*21*). Because of the increase in skin and soft tissue infections, standard precautions, including use of gloves, are likely warranted when dealing with all skin and soft tissue infections in outpatient clinics and acute-care facilities. Contact precautions, including use of gowns and gloves, should be implemented for all wound care in acute-care facilities, and institutional programs to enhance antimicrobial drug stewardship should be implemented. Programs to increase community awareness to control spread of infections and initiatives to reduce inappropriate use of antimicrobial drugs should also be implemented, especially in institutions that are focal institutions such as daycare centers, schools, and prisons, as well as in high-risk groups such as immunodeficient persons, children, and elderly persons. Clinicians should be aware of the magnitude of the issue and consider MRSA a highly likely cause of skin and soft skin tissue infections, even in areas where the prevalence of MRSA is believed to be low.

Previous hospitalization has been associated with community MRSA carriage (*22*), and some recent studies have suggested that MRSA infection rates in the community are positively correlated with *S*. *aureus* infection rates in the hospital (*23*,*24*). Although a recent study suggests that community-associated MRSA is causing hospital-acquired MRSA (*25*), it is unclear from our study whether community-associated MRSA is responsible for increasing rates of nosocomial MRSA or the other way around. In all likelihood, MRSA is spreading in hospitals and communities and complicating efforts to prevent infections in hospitalized patients. Regardless, our findings demonstrate that recent reports of localized increases in community-acquired MRSA (*7*,*26*–*28*) are part of a larger trend of MRSA becoming rapidly endemic in communities all over the United states, emulating the wave-like pattern of emerging resistance to penicillin in the middle of the 20th century (*5*).

Hospital-acquired infections from all causes are estimated to cause >90,000 deaths per year in the United States and are the sixth leading cause of death nationally. Nosocomial infections increase patient illness and the length of hospital stays. The direct cost has been estimated to be >$6 billion (inflation adjusted) (*29*); costs of longer inpatient visits are shared by hospitals. The increasing trend in hospitalizations associated with *S*. *aureus* infections has considerable cost implications for the healthcare system, including costs when community-associated infections require hospitalization and the additional expenses from associated nosocomial infections.

Antimicrobial drug–resistant infections impose even greater costs than susceptible infections. Several studies have estimated that antimicrobial drug–resistant infections increase death, illness, and direct costs by 30%–100% (*30*). Estimates of the excess cost of an infection with MRSA compared with an infection with methicillin-sensitive *S*. *aureus* range from ≈$3,000 to $35,000 (*31*–*33*). This suggests that MRSA cost the healthcare system (patients and hospitals) an extra $830 million–$9.7 billion in 2005, even without taking into account indirect costs related to patient pain, illness, and time spent in the hospital.

Another important implication of our analysis is that the increasing incidence of MRSA in hospitalized patients, whether the infection was acquired in the hospital or the community, is likely to increase the demand for vancomycin. Despite several new (daptomycin, linezolid, tigecycline) and old (trimethoprim-sulfamethoxazole, clindamycin) antimicrobial drugs available for treatment of MRSA infections, vancomycin has remained the first-line drug for treating MRSA (*12*,*34*). This pattern has broad implications for the future control of MRSA as well as other pathogens. *S*. *aureus* infections resistant to vancomycin are already emerging (*35*), and vancomycin-resistant enterococci are already a major problem in hospitals. Vancomycin use should be restricted to methicillin-resistant *S*. *aureus* infections and used only for MRSA infections in situations where other drugs are not appropriate.

Our analysis has some limitations. First, it was restricted to the incidence of disease associated with acute-care management within the hospital setting. Recent reports suggest that MRSA has been increasing in outpatients (*36*,*37*). Thus, our results represent only a part of the problem, although hospitalizations outweigh outpatient visits by ≈4 to 1.

Second, NHDS data enables the coding of only 7 diagnosis codes; hospital information systems typically include 15–20 diagnosis codes for each admission (*38*). Thus, additional diagnoses in which *S*. *aureus* played a role may have been excluded. Errors in ICD coding when transcribing from doctors’ discharge summaries are another potential source of bias, as is the possibility that multiorgan failure, an end stage of sepsis, was coded as septicemia. One study concluded that the positive predictive value of the 038 code on NHDS records to predict sepsis was 88.9%–97.7%, depending on the criteria, and the negative predictive value was 80.0% (*39*). The authors of another study that examined whether sepsis was coded correctly on hospital bills concluded that strict reliance on administrative data may be prone to bias because only 75.4% of sepsis cases were accurately coded (*38*). Thus, our results may be an underestimate of the true effect, although trends are likely robust to coding errors.

Third, TSN data provide information concerning only the site of isolate collection and not the infection. Thus, some isolates from blood or the lung area may not be associated with septicemia or pneumonia, respectively. In addition, the code for *S*. *aureus* septicemia was given priority over the other more site-specific codes; this could have affected the estimates of MRSA infections. However only a limited number of records had overlapping codes.

**Figure 4:**
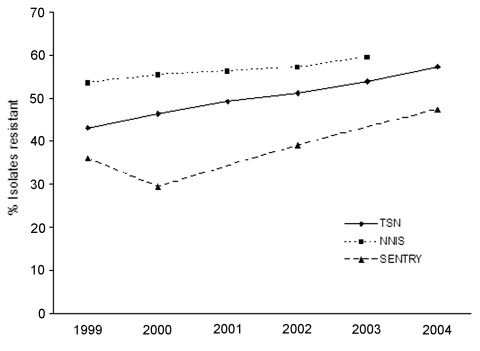
Percentage of *Staphylococcus aureus* isolates resistant to methicillin in national surveys, United States, 1999–2004. TSN, The Surveillance Network (data include hospital infections); NNIS, National Nosocomial Infections Surveillance System (data include only intensive care units); SENTRY, includes only skin and soft tissue infections.

Fourth, although the 2 data sources from TSN and NHDS used in this article are nationally representative, they may not represent a stratified random sample of hospitals by type and region. However, the trends are likely robust enough to avoid bias. In addition, the percentage of *S*. *aureus* isolates resistant to methicillin reported in the TSN database has increased similar to that reported by other national studies (Figure 4). Finally, our estimates of the number of hospitalizations and deaths are associated with, but cannot be directly attributed to, *S*. *aureus* and MRSA because NHDS does not report the immediate cause of death, and older, sicker patients are more likely to contract a nosocomial infection (*40*).

Our findings suggest that *S*. *aureus* and MRSA should become a national priority for disease control. Possible responses include expanding national surveillance or reporting requirements for *S*. *aureus* and MRSA infections, more research to quantify the relative importance and interaction between community- and healthcare-associated colonization and infection, improved investments in hospital-infection control, and greater public investment to support research and development of an *S*. *aureus* vaccine.
